# MYC and integrins interplay in colorectal cancer

**DOI:** 10.18632/oncoscience.293

**Published:** 2016-02-13

**Authors:** Salah Boudjadi, Jean-François Beaulieu

**Affiliations:** Laboratory of Intestinal Physiopathology, Department of Anatomy & Cell Biology, Faculty of Medicine and Health Sciences, Université de Sherbrooke, Sherbrooke, QC, Canada, J1H 5N4

**Keywords:** MYC, integrin, colorectal cancer

The proto-oncogene MYC is one of several well-known transcription factors involved in the genesis and progression of many types of cancer acting as a main regulator of the expression of genes involved in cell proliferation, invasion, apoptosis, metabolism, DNA repair and protein synthesis [1, 2]. Different mechanisms are involved in the deregulation of MYC expression in cancer including gene mutation and amplification as well as upregulation by activated upstream pathways namely WNT/APC/β-catenin and receptor tyrosine kinase(RTK)/RAS/MEK/ERK pathways [1], both frequently activated in colorectal cancer (CRC). Defects in the former pathway prevent β-catenin phosphorylation by GSK3β kinase allowing β-catenin translocation to the nucleus where it can enhance MYC transcription by binding to TCF on the MYC promoter. GSK3β inhibition also prevents MYC phosphorylation at threonine 58 preventing its degradation while MYC phosphorylation on serine 62 by ERK favors its stabilization.

These two sets of events illustrate the potential regulation of MYC expression in CRC cells.

The regulation of gene transcription by MYC has also been reviewed in detail [1, 2]. Essentially, MYC functions as a transcriptional regulator in association with its partner MAX; the MYC/MAX heterodimer binds to DNA in a sequence-specific manner to activate transcription. Studies over the last decade have identified and characterized several additional partners defined as the MYC/MAX/MAD network that cooperate in the modulation of MYC transcriptional activity [2]. The MYC/MAX heterodimer binds to the consensus sequence 5′-CANNTG-3′ (E box) to regulate the transcription of genes [1]. The occurrence of canonical MYC E-box motifs is high in the human genome. While they can be bound by other E box transcription factors in non-proliferating cells for basal cell metabolism, binding of the E box by MYC/MAX appears to be favoured in cells displaying high levels of MYC, leading to a change in metabolism [1].

Integrins are among the genes regulated by MYC. For instance, *ITGA6, ITGB1* and *ITGB4* promoters contain a canonical E box binding site for MYC. In the mouse skin, MYC represses *Itga6*, *Itgb1* and *Itgb4* expression via the formation of a complex with MIZ1, which mediates MYC repression of gene expression [3]. However, in CRC cells, MYC positively regulates *ITGA6* (Groulx *et al*., unpublished) and *ITGB4* [4] expression supporting the notion that MYC transcriptional activity is cell context-dependent.

A recent study from our group identified another integrin subunit upregulated in colorectal cancer and in colorectal tumour cell lines: ITGA1 [5]. In order to find whether ITGA1 is regulated in the CRC context, *in silico* analysis of the *ITGA1* promoter was performed and led to the identification of two E box-like response elements CAAGTG and CAGATG, which were found to be functional, as demonstrated by promoter reporter studies [5]. Indeed, *in cellulo* experiments showed that forced expression of MYC enhances activity of the *ITGA1* promoter while co-expression of MYC and MAD or disruption of one of the response elements identified on the promoter reduces it. The functionality of this link was confirmed by the binding of MYC to the *ITGA1* promoter in the native chromatin of CRC cells [5]. Furthermore, pharmacological MYC inhibition or shRNA knockdown resulted in a drastic reduction in ITGA1 expression at both the protein and mRNA levels in three distinct CRC cell lines. The functional relevance of these data suggesting that MYC regulates ITGA1 expression at the transcriptional level was strengthened by the finding that MYC and ITGA1 protein expressions are found to be correlated in more than 72% of the colorectal tumour samples analyzed [5].

**Figure 1:**
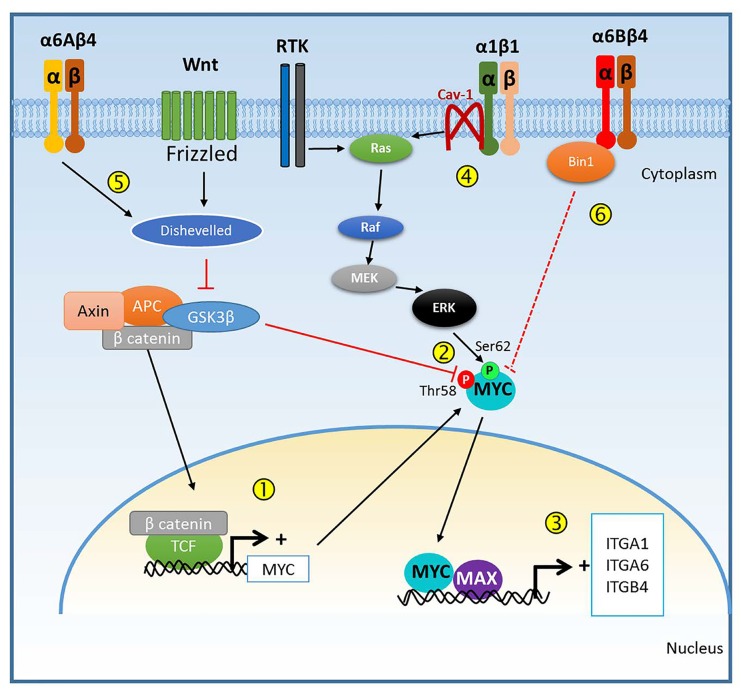
MYC and integrin mutual regulation Diagram summarizing the possible interactions between MYC expression/activity, and some of the integrins whose expression is regulated by the transcriptional activity of MYC.

In the CRC context, as summarized in Figure 1, deregulation of the WNT/APC/β-catenin and RTK/RAS/MEK/ERK pathways both enhance MYC expression and protein stabilization (Figure 1, steps 1 and 2) [1]. MYC can then dimerize with MAX to bind E boxes to regulate the transcription of many genes [2] among which are the integrin subunit mRNAs *ITGA1, ITGA6* and *ITGB4* (Step 3) [4, 5] resulting in the up-regulation of integrin α1β1 and α6β4 in its α6Aβ4 form, which can further activate the RAS/MEK/ERK pathway (Step 4) and promote β-catenin signaling by stabilizing the GSK3β inhibitor Dishevelled (Step 5) [6] to enhance MYC expression (Steps 1-2). This suggests the existence of a potential positive feedback loop for sustaining MYC and integrin activity and strengthens their involvement in cancer progression. In this context, it is noteworthy that the α6Bβ4 integrin, the other form of α6β4 that is not increased in CRC cells [6, 7], was found to inhibit CRC cell proliferation and MYC activity, an effect that was explained by the fact that the α6B subunit tail can bind to the MYC inhibitor protein bridging integrator-1 (BIN1) [7] (Step 6). Further understanding of this interplay between MYC and some of these integrins should lead to the development of new therapeutic strategies for specifically targeting MYC for the design of more efficient CRC treatments.
